# MR Imaging Features of Juvenile Pilocytic Astrocytoma in the Suprasellar Region: A Study on 11 Patients

**DOI:** 10.2174/0115734056347108250318083203

**Published:** 2025-04-17

**Authors:** Xiaocai Zhang, Hongyue Tao, Zhenqing Liu, Zidong Zhou, Li Huang, Guangbi Song

**Affiliations:** 1 Department of Radiology, Luoping County People's Hospital, Yunnan Province, China; 2 Department of Radiology, Huashan Hospital, Fudan University, Shanghai, China; 3 Department of Radiology, Guangzhou Women and Children's Medical Center, Guangzhou, China

**Keywords:** Juvenile, Suprasellar region, Pilocytic astrocytoma, Magnetic resonance imaging, T2W images, Apparent diffusion coefficient, Cerebrospinal fluid

## Abstract

**Objective:**

This study aimed to characterize the magnetic resonance imaging (MRI) findings of juvenile suprasellar pilocytic astrocytoma (PA) in a sample of 11 children and help neuroradiologists preoperatively differentiate PAs from other suprasellar tumors.

**Methods:**

Eleven consecutive children with pathologically confirmed suprasellar PAs in our hospital from May 2015 to November 2021 were enrolled in this study. The clinical data and preoperative MR images were retrospectively reviewed. MRI included T1-weighted imaging (T1WI), T2-weighted imaging (T2WI), fluid-attenuated inversion recovery (FLAIR), and postcontrast T1WI. Six patients underwent diffusion-weighted imaging (DWI). The location, signal features, enhancement pattern, and apparent diffusion coefficient (ADC) of the lesions on MRI were evaluated. The clinical status of the patients 3 years after surgery was noted.

**Results:**

The 11 suprasellar PAs were mainly located around the optic chiasma and hypothalamus and invaded adjacent structures. The lesions showed hyperintensity or slight hyperintensity on T2WI and hypointensity on T1WI. Among the 11 patients, 5 had solid tumors with homogeneous enhancement, one had a solid tumor with heterogeneous enhancement, and five had cystic and solid tumors with heterogeneous enhancement. Cerebrospinal fluid (CSF) dissemination foci were observed in 4 patients. The solid components of the lesions were hypointense or isointense on DWI, with high ADC values at a mean of 1.77±0.36 ×10^-3^ mm^2^/s. Gross total resection was achieved in only one patient (9.1%), and 10 (90.9%) were subtotally resected. Five patients died during the follow-up period, and the 3-year survival rate was 54.5%.

**Conclusion:**

Juvenile suprasellar PAs are characterized by a solid and intermixed cystic and solid appearance, hyperintensity on T2W images, obvious enhancement of the solid component, and relatively high ADC values.

## 1 INTRODUCTION

Pilocytic astrocytoma (PA) was first described by Harvey Cushing in 1931 [1]. It is classified as a grade I astrocytoma according to the 2021 5th edition of the World Health Organization (WHO) Classification of Tumors of the Central Nervous System (CNS) [2]. Among all primary central nervous system tumors, PA represents 2.3% of cases in the general patient population and accounts for 8.0-23.5% of CNS tumors in pediatric patients [3, 4]. Typically benign and well-defined, PAs seldom infiltrate nearby brain tissues or progress to aggressive forms, like anaplasia, meningeal dissemination, or local recurrence [5]. Achieving complete surgical removal of PAs often results in a cure, boasting impressive 10-year survival rates of up to 95% [6].

PAs are predominantly found in the cerebellum, with rare occurrences in the suprasellar region [7, 8]. When PAs arise in the suprasellar region, they often stem from the optic chiasma, hypothalamus, or base of the third ventricle, posing challenges for complete resection and resulting in less favorable outcomes [9]. Furthermore, due to the presence of subtle and atypical clinical manifestations in some pediatric patients with suprasellar PAs, misdiagnosis is common, particularly in young children.

Preoperative accurate diagnosis of juvenile suprasellar PAs may be helpful and crucial in surgical planning and prognosis prediction. Therefore, in this study, we analyzed clinical and magnetic resonance imaging (MRI) data from 11 individuals diagnosed with this uncommon tumor, conducted a thorough literature review, and pinpointed imaging characteristics that offer the highest specificity in detecting juvenile suprasellar PAs.

## 2 MATERIAL AND METHODS

### 2.1 Patients

This study was performed according to regulations of the ethics committee for human research at our hospital (Luoping County People's Hospital, China; no. LPLL-2021-027), and written informed consent was obtained from the patients’ parents. Patients with histologically proven suprasellar PAs following surgery at our hospital between May 2015 and November 2021 were retrospectively analyzed. The clinical information, MRI, and pathologic findings of these patients were reviewed. The inclusion criteria were the presence of suprasellar PAs confirmed by pathology, complete medical records, and preoperative head examination on MRI with enhancement. The exclusion criteria were patients with suprasellar lesions other than PAs confirmed by pathology, incomplete medical records or missing preoperative MRI with enhancement, and patients lost to follow-up. Twenty-four young children with suprasellar PAs were confirmed by pathology; ten of them were without enhanced MRI at our hospital and three lost to follow-up, and thus were excluded. Finally, 11 patients were included in our study. Clinical data, such as age, sex, symptoms, and duration of symptoms, were reviewed. The clinical status of the patients at the end of the 3-year follow-up period was noted.

### 2.2 Imaging Technique and Evaluation

MR images were acquired using a Siemens 3.0-T scanner (Siemens Skyra) with an MRI phased array head coil. Before the MRI examination, the children were given oral chloral hydrate for sedation (0.5 ml/kg), and an MRI was performed after they fell asleep. The following scanning sequences were used: sagittal and axial T1-weighted imaging (T1WI) [repetition time (TR) 250 milliseconds (ms), echo time (TE) 2.9 ms], axial T2-weighted imaging (T2WI) (TR 4330 ms, TE 109 ms), fluid-attenuated inversion recovery (FLAIR) (TR 8000-9000 ms, TE 111-120 ms), and contrast-enhanced sagittal and axial T1WI after the intravenous injection of gadopentetate dimeglumine (Gd-DTPA, Magnevist; Bayer Schering Pharma, Berlin, Germany) at a dose of 0.1 mmol/kg. In addition, diffusion-weighted imaging (DWI, single shot, spin-echo, and echo-planar imaging sequences) (TR 3500 ms, TE 98 ms, b=0 and 800 seconds/mm2) was performed on 6 patients. All sequences were acquired according to the following parameters: field of view 230 mm×230 mm, layer thickness 5 mm, and interslice spacing 1 mm.

All MR images were retrospectively analyzed by 2 experienced pediatric neuroradiologists (with 22 and 13 years of experience, respectively) who were blinded to the histological results. The tumor location, invasion of adjacent structures, size, shape, signal characteristics, enhancement pattern, dilatation of the supratentorial ventricle, and presence of cerebrospinal fluid (CSF) dissemination were evaluated. ADC values were calculated on the ADC map using the Functool software program (Siemens Skyra workstation). The regions of interest (ROIs) were manually placed in the solid portion of the tumors. According to their postcontrast T1WI characteristics, the lesions were classified into 3 patterns: solid tumors with homogeneous enhancement, solid tumors with heterogeneous enhancement, and solid and cystic tumors with heterogeneous enhancement. The descriptive statistics for the continuous variables are reported as the arithmetic mean value and standard deviation (SD).

### 2.3 Pathological and Immunohistochemical Examinations

All patients underwent surgical resection. Histological examinations were then performed on all 11 specimens. Surgical specimens were fixed in 10% neutral-buffered formalin. The histomorphological features were observed using hematoxylin and eosin (H&E)-stained sections by two neuropathologists. In a subset of patients, immunohis-tochemistry (IHC) was performed for GFAP, SYN, Olig2, S-100, EMA, NeuN, CD34, Ki-67, IDH1 mutations, and P53.

## 3 RESULTS

### 3.1 Clinical Data

Table **1** summarizes the clinical features of 11 patients with suprasellar PAs. Five patients were boys, and 6 were girls. Their age ranged from 5 months to 72 months, with a mean age of 35 months. The main clinical symptoms included emesis (27.2%) (3/11), nystagmus (18.2%) (2/11), retarded development (18.2%) (2/11), and headache (18.2%) (2/11). One patient had symptoms of polydipsia and polyuria. One patient with no specific symptoms was diagnosed with PA during a healthy head MRI examination due to the neurofibroma of his mother. No patients received postoperative adjuvant radiotherapy or chemotherapy. The mean follow-up was 22 months (range: 6 to 36 months). Five patients died, yielding an overall survival of 55% at the 3-year follow-up after surgery.

### 3.2 MRI Findings

Table **2** summarizes the MRI features of 11 juveniles with suprasellar PAs.

Location: The location and invasion characteristics of adjacent structures were as follows: 11 PAs were mainly located around the optic chiasma in the suprasellar region; 7 PAs extended into the sellar region and invaded the pituitary stalk, but the pituitary gland was visible; 1 PA invaded the posterior pituitary lobe, leading to the disappearance of the posterior pituitary, which showed hyperintensity on T1WI; 9 PAs involved the hypothalamus; 6 PAs involved the optic nerve (4 PAs involved bilateral optic nerve involvement, 2 PAs involved left optic nerve involvement); 3 PAs involved the internal carotid artery; 3 PAs involved the anterior cerebral artery; 1 PA involved the oculomotor nerve; 1 PA involved the middle and posterior cerebral artery; 1 PA involved the posterior communicating artery; 1 PA involved the left temporal lobe; and 1 PA involved the gray tubercle.

Size and shape: The maximum diameter of the 11 suprasellar PAs ranged from 2.9 cm to 9.1 cm, with an average of 5.1±1.8 cm. Three patients had lobulated lesions, and 8 patients had irregular lesions.

Signal characteristics: Five patients showed hyperintensity on T2W images, which was close to the signal intensity of CSF, and hypointensity on T1W images. Five patients showed slight hyperintensity on T2WI, among whom 4 had hypointensity on T1WI and 1 had slight hyperintensity on T1WI. One patient showed mixed signal intensity on T1WI and T2WI due to hemorrhage in the tumor.

Enhancement characteristics: Five solid tumors were strongly enhanced. One patient had a solid tumor with heterogeneous enhancement (multiple necrotic foci in the center). Five patients had solid and cystic tumors, of which the solid component was significantly enhanced, while the cystic component had no enhancement or only mild enhancement of the cystic wall.

Peritumoral edema: None of the 11 patients had peritumoral edema. Eight patients had dilation of the supratentorial ventricle.

CSF dissemination foci: Four patients had multiple dissemination foci of different sizes in the cerebral-based cistern (including the cisternal annulus, cisternal anterior pontine, and cisternal greater occipitalis) and on the leptomeninges of the medulla oblongata and cervical cord.

DWI: Suprasellar PAs present hypointensity or isointensity on DWI. The ADC values of the solid components of the tumors were measured in 6 patients. The ADC values were high, with a mean of 1.77±0.36 ×10^-3^ mm^2^/s. Fig. (**1**) shows the suprasellar PA of a 5-month-old boy.

### 3.3 Pathological and Immunohistochemical Examinations

Gross observation revealed six specimens to be solid tumors (1 with micro necrotic foci), and 5 to be solid and cystic. The six solid tumors had a fish-like appearance, with medium hardness and abundant blood supply. The solid-cystic tumors of five patients were grayish white, with vesicles and pale yellow vesicle fluid.

#### 3.3.1 Histological features

The majority of PAs showed moderate cellularity and microcystic changes. The tumor cells exhibited bipolar differentiation, with dense spindle-shaped cells in some areas and loose spindle-shaped cells in other areas. Rosenthal fibers and eosinophilic granular bodies were present in the majority of the tumors. Calcification, inflammation, and foci of chronic hemorrhage were rarely observed.

Immunohistochemical examinations (Table **3**): GFAP, SYN, S-100, Ki-67, and Olig2 were positive in the majority of 11 patients. EMA was positive in 4 patients and negative in 5 patients. NeuN was positive in 2 patients and negative in 6 patients. IDH was positive in 1 patient and negative in 3 patients. CD34 was positive in 5 patients and negative in 4 patients. p53 expression was negative in 2 patients and positive in 1 patient.

### 3.4 Follow-up

Gross total resection was achieved in only one patient (9.1%), and 10 patients (90.9%) were subtotally resected. The mean follow-up period for all patients was 22 months (range: 6 to 36 months). Five patients died during the postoperative period, and the 3-year survival rate was 54.5%.

## 4 DISCUSSION

PA represents a distinctive histologic subtype of astrocytoma that predominantly affects children and adolescents. Its occurrence in the suprasellar region is relatively uncommon. PA is typically observed in individuals under the age of 20, and its incidence declines with increasing age [10]. In our study, the ages of the 11 patients ranged from 5 to 72 months. There are different reports on the age of PA occurrence. Komotar *et al*. [11] reported 42 cases of PA, with an average age of 58 months. However, the average age of 11 patients with suprasellar PAs in our study was 35 months, among whom 5 were less than 2 years old (accounting for 45.4%). The age of onset was significantly younger than that in the above studies, possibly because our patients were recruited from a specialized hospital for children. There was no sex difference (male/female ratio of 5:6) in the incidence of PAs in our study, being consistent with previous studies [1].

MRI serves as the imaging modality of choice for identifying and characterizing suprasellar tumors. On MRI scans, the predominant imaging pattern of suprasellar PA typically manifests as a solid or solid-cystic mass. Among the 11 patients with suprasellar PAs in our study, 3 patients with solid tumors showed hypointensity on T1WI and hyperintensity on T2WI, while the solid component of the remaining 8 patients showed slight hyperintensity on T2WI, which was significantly greater than that of gray matter. The cystic portion of 5 solid-cystic tumors showed hypointensity on T1WI and hyperintensity on T2WI, which was close to the CSF. The signal intensity on T2-weighted images of suprasellar PAs exhibited variability due to the uneven biphasic distribution of tumor cells, with some primarily comprising pilocytic components and others mainly composed of glial components. Arai *et al*. [12] proposed that the hyperintensity of the solid component of PAs on T2-weighted images served as a more effective distinguishing feature from other tumors.

After contrast, there were 3 predominant enhancement patterns of suprasellar PAs in our study: (1) a solid tumor with homogeneous enhancement (27.3%), (2) a solid tumor with heterogeneous enhancement (27.3%), and (3) a solid-cystic tumor with heterogeneous enhancement (45.5%). Notably, the solid components of all 11 tumors exhibited marked enhancement, which was attributed to the unique vascular wall of PAs, characterized by endothelial cells with open tight junctions and fenestrae, allowing for contrast extravasation [13]. PA tumor cells have the capacity to secrete cyst fluid, leading to the aggregation and formation of cysts within the tumor. The cystic component of a PA is known to contain abundant vascular growth factors, which in turn promote vascular proliferation, consequently contributing to the substantial enhancement of the solid component of the tumor [7]. In our case series, 5 patients exhibited prominent large cysts located at the periphery of the mass.

The majority of the solid components of PAs appeared isointense or hypointense on DWI. The average ADC of the tumors exceeded that of the white matter, suggesting unhindered diffusion of water molecules among tumor cells. These findings align closely with those reported in a prior study [14].

Hemorrhage in PAs has been described as an uncommon imaging feature that was observed in only one patient in our series. Contrary to earlier beliefs, White *et al*. [15] reported intratumoral hemorrhage in 8% of PAs, indicating a higher prevalence than that previously recognized. While hemorrhage may not be a common presentation, its presence should not preclude the diagnosis of PA.

While CSF dissemination is uncommon in PAs, isolated cases of PAs spreading to the subarachnoid spaces have been reported [16-18]. Previous studies indicate that less than 5% of PAs are associated with CSF dissemination [19]. It has been hypothesized that malignant transformation, cellular anaplasia, or natural history contribute to the spread of the tumor. However, in this study, we identified 4 patients (36.4%) with PAs with CSF dissemination, being more than the proportions in the abovementioned reports. Primary suprasellar PAs have the potential to invade the subarachnoid spaces or deposit tumor cells in the lacunae of the pia mater via intravascular or perivascular routes, ultimately leading to leptomeningeal dissemination through the CSF [18, 20, 21].

PAs should be distinguished from other common diseases in the suprasellar region in children.

Ameloblastic craniopharyngiomas (ACPs): ACPs, like PAs, are relatively prevalent in children and are typically cystic or solid-cystic lesions. They often exhibit eggshell-like calcifications on CT scans, hyperintense cystic components on T1WI, heterogeneously mixed signal intensity on T2WI, and usually elevated ADC values. Following contrast administration, ACPs demonstrate significant enhancement in the solid portion and eggshell-like enhancement of the cystic wall [22]. Xu *et al*. [22] suggested that the cut green pepper sign, an irregular rim of peripheral enhancement of a PA, is useful for differentiating PAs from ACPs in the suprasellar region. Surgical resection of the tumor is the preferred treatment for suprasellar ACP. The prognosis of suprasellar ACP is usually good, especially after total tumor resection [23]. Germinoma: Patients with germinomas in the suprasellar region commonly present with diabetes insipidus. Laboratory investigations may reveal elevated levels of alpha-fetoprotein (AFP) and human chorionic gonadotropin (HCG). Germinomas typically display iso- or low-intensity signals on T1WI, slight hyperintensity on T2WI, and uniform and pronounced enhancement post-contrast. They are characterized by pituitary stalk thickening, easy cerebrospinal fluid dissemination, and concurrent involvement of the pineal region [24]. In addition, the relatively low ADC values of the germinomas can aid in distinguishing them from PAs. Germinomas are sensitive to radiotherapy and chemotherapy, which are the main treatment methods besides surgical resection. Pilomyxoid astrocytoma (PMA): Originally, PMA was designated a WHO grade II tumor in the CNS because of its more aggressive nature and higher rates of recurrence and CSF spread [2]. PMAs are more likely to occur in infants and young children, especially in the suprasellar region [25]. Typically, PMAs present as large complex solid-cystic tumors with MRI features of T2 hyperintensity and elevated ADC values, reflecting a higher proportion of the myxoid matrix [26]. In an observation, after contrast, 48% of tumors showed heterogeneous rim enhancement, 43% showed uniform enhancement, and 9% showed no enhancement [27]. Lee *et al*. [28] proposed that the enhancement of solid portions in some PMAs was not as obvious as that in PAs, and even some PMAs were not enhanced. Overall, younger age, a more frequent occurrence in the suprasellar area, a solid mass containing a non-enhancing portion, and more frequent leptomeningeal dissemination are helpful differentiating features of PMAs from PAs [28]. The preferred treatment for PMAs is surgical resection of most tumors, followed by adjuvant chemotherapy and radiotherapy as needed after surgery. PMAs usually have a worse prognosis than PAs, with a higher recurrence rate and shorter overall survival after treatment [29]. The typical MRI findings of these tumors that should be differentiated from PAs are shown in Fig. (**2**). The treatment and prognosis of these tumors in the suprasellar region are different. Therefore, early and accurate diagnosis is crucial to enable timely and suitable treatment of patients.

Histologically, PAs are characterized as benign tumors with low to moderate cellularity, exhibiting loosely textured areas comprising multipolar cells, microcysts, and eosinophilic granular bodies. These tumors also present densely fibrillated regions rich in Rosenthal fibers, featuring cells with elongated bipolar processes and cytologically bland nuclei [9]. The unique histopathological changes seen in PAs reflect their typically indolent nature, occasionally demonstrating spontaneous degenerative behavior [30]. AlRayahi *et al*. [31] documented that the immunohistochemistry profile of PAs commonly exhibited positivity for GFAP, S-100, and Olig2 markers, while negative for IDH and p53 mutations. In our study, IHC analysis of PAs revealed GFAP, S-100, and Olig2 to be positive in 11 patients; SYN was also positive in most patients, IDH was positive in one patient, and p53 was positive in 2 patients. PMAs are identified by monomorphously organized little bipolar cells with spindle-shaped nuclei and a considerable myxoid background, without Rosenthal fibers, mitoses, or eosinophilic granular bodies [29]. The immunohistochemical features of PMAs are practically close to those of PAs [25].

Surgery with gross total resection is the preferred treatment, with a 10-year survival rate of 95% [5, 6]. However, resection of the suprasellar PA in children is difficult due to its intricate location, extensive tumor involvement, adherence to adjacent tissues, and complex surrounding anatomical structures. In our study, complete resection was achieved in only one patient, and partial resection was performed in the other 10 patients, resulting in a poor prognosis (a 3-year survival rate of 54.5%).

Our study involved several limitations. First, this was a retrospective study, and there may exist selection bias and information bias. Second, the number of patients was limited, which may limit the statistical significance of the results, and disable the multivariate analysis to explore the relationship between imaging features and prognosis. Third, we included only 6 patients with PAs for whom DWI data were available, further limiting the comprehensive analysis of ADC values.

## CONCLUSION

In summary, suprasellar PAs are relatively rare central nervous system tumors in young children. Juvenile suprasellar PAs are characterized by a solid and intermixed cystic and solid appearance, hyperintensity on T2W images, obvious enhancement of the solid component, and relatively high ADC values. Understanding the imaging characteristics is helpful and crucial for preoperative accurate diagnosis of suprasellar PAs. However, future larger studies with an emphasis on DWI of suprasellar PAs are needed to validate these findings. Multivariate analysis should also be performed to explore the relationship between imaging features and prognosis.

## Figures and Tables

**Fig. (1):**
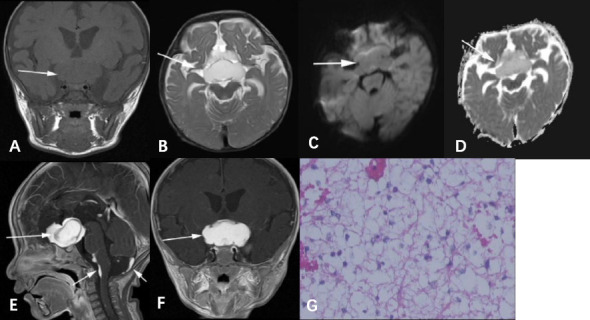
A suprasellar PA in a 5-month-old boy. The tumor (arrow) was lobular and irregular in shape. It showed homogeneous hypointensity on the coronal T1WI (**A**), homogeneous hyperintensity on axial T2WI (**B**), isointensity on DWI (**C**), hypointensity on ADC map (**D**), and significant enhancement on the postcontrast T1WI (**E** and **F**). There were multiple disseminated foci on the surface of the pons and medulla oblongata (short arrows). **G**. H-E staining (original magnification ×100) revealed loose and multipolar tumor cells with microcapsule structure and no obvious atypia.

**Fig. (2):**
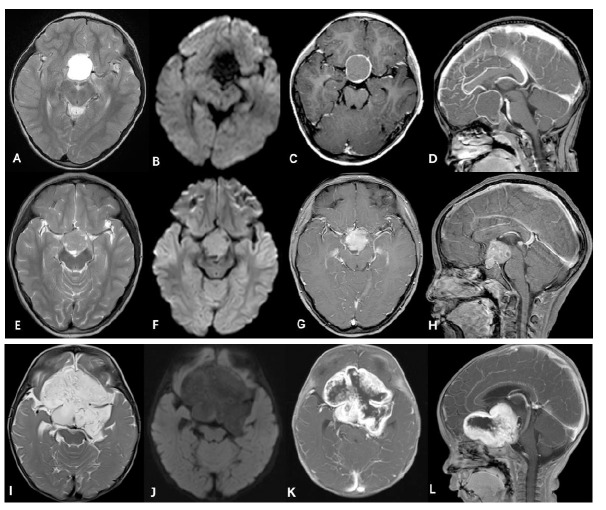
A pathologically confirmed ACP in a 6-year-old girl. It was a cystic lesion, exhibiting hyperintensity on T2WI (**A**) and no restriction on DWI (**B**). Following contrast administration, the ACP demonstrated significant ring-like enhancement in the cystic wall (**C** and **D**). A pathologically confirmed suprasellar germinoma in an 11-year-old girl. The tumor displayed slight hyperintensity on T2WI (**E**), typical hyperintensity on DWI (**F**), and uniform and pronounced enhancement post-contrast (**G** and **H**). A pathologically confirmed suprasellar PMA in an 8-month-old boy. It presented as a large complex solid-cystic tumor with hyperintensity on T2WI (**I**) and no restriction on DWI (**J**). After contrast (**K**, **L**), the tumor showed heterogeneous rim enhancement.

**Table 1:** The clinical data of the 11 children with suprasellar PA.

Case No.	Gender (M/F)	Age (month)	Duration of Symptoms (Month/Day)	Symptoms	Extent of Resection	Time to Follow-up after Surgery (Month)	Outcome
1	M	5	1m	Emesis	Subtotal resection	6	Died
2	M	48	3d	Mother had a history of NF1, physical examination	Subtotal resection	36	Alive with tumor
3	M	36	12m	Strabismus, developmental lag	Subtotal resection	30	Lost to follow-up
4	M	23	15d	Emesis	Subtotal resection	6	Died
5	F	48	15d	Enuresis, polyuria	Subtotal resection	36	Alive with tumor
6	F	10	5m	Nystagmus	Subtotal resection	6	Died
7	F	72	5m	Headache	Subtotal resection	36	Alive with tumor
8	F	13	1m	Emesis	Subtotal resection	6	Died
9	F	11	3m	Developmental lag	Subtotal resection	6	Died
10	M	72	3d	Headache, lethargy	Gross total resection	36	Alive
11	F	48	10d	Nystagmus	Subtotal resection	36	Alive with tumor

**Table 2:** The MRI features of the 11 children with suprasellar PA.

Case No.	Maximum Diameter (cm)	Location and Invasion	Signal Intensity on T1WI/T2WI	Enhancement Patterns	Dilatation of Supratentorial Ventricle	CSF Dissemination	ADC Values (10^-3^mm^2^/s)
1	4.1	Optic chiasma, left optic nerve	Low/ slightly high	Solid homogeneous	+	+	1.78
2	2.9	Optic chiasma, hypothalamus	Low/ slightly high	Solid homogeneous	_	-	/
3	4.1	Optic chiasma, hypothalamus, pituitary stalk	Sightly low/ slightly high	Solid homogeneous	+	-	1.28
4	5.4	Optic chiasma, bilateral optic nerve, pituitary stalk	Low/high	Solid homogeneous	+	-	1.92
5	3.9	Optic chiasma, hypothalamus, pituitary stalk, posterior pituitary lobe	Low/ slightly high	Solid-cystic heterogeneous	_	+	/
6	7.2	Optic chiasma, bilateral optic nerve, hypothalamus, pituitary stalk, internal carotid artery, anterior cerebral artery	Low/ high	Solid-cystic heterogeneous	+	-	1.78
7	3.9	Optic chiasma, hypothalamus	Low/ high	Solid-cystic heterogeneous	_	+	/
8	5.5	Optic chiasma, bilateral optic nerve, hypothalamus, pituitary stalk	Low/ high	Solid homogeneous	+	-	/
9	4.5	Optic chiasma, hypothalamus	Low/ high	Solid-cystic heterogeneous	+	-	2.35
10	5.8	Optic chiasma, left optic nerve, hypothalamus, pituitary stalk, internal carotid artery	Mixed /mixed	Solid-cystic heterogeneous	+	-	1.53
11	9.1	Optic chiasma, bilateral optic nerve, hypothalamus, pituitary stalk, oculomotor nerve, internal carotid artery, posterior cerebral artery, posterior communicating artery, left temporal lobe, gray tubercle	Low/ slightly high	Solid-cystic heterogeneous	+	+	/

**Abbreviations:** PA = pilocytic astrocytoma; T1WI = T1-weighted imaging; T2WI = T2-weighted imaging; ADC = apparent diffusion coefficient; CSF = Cerebrospinal fluid.

**Table 3:** Immunohistochemical examinations of the 11 suprasellar PA.

Case No.	GFAP	SYN	Olig2	S-100	EMA	NeuN	CD34	Ki-67	IDH1	P53
1	+	+	/	+	-	-	-	3%+	/	/
2	+	+	+	+	-	/	+	1%+	-	+
3	+	-	/	+	/	-	/	1%+	/	/
4	+	/	+	+	/	/	/	1%+	/	/
5	+	+	+	+	-	-	-	5%+	/	/
6	+	+	+	+	+	-	+	1%+	/	/
7	+	+	/	+	-	-	-	1%+	/	/
8	+	+	+	+	+	+	+	10%+	-	/
9	+	/	+	+	+	/	+	1%+	+	/
10	+	-	+	+	-	-	+	1%+	/	+
11	+	+	+	+	+	+	-	1%+	-	-

**Abbreviations**: GFAP=glial fibrillary acidic protein, Olig2=oligodendrocyte transcription factor,SYN=Synaptophysin, EMA=Epithelial Membrane Antigen, NeuN=Neuronal Nuclear Antigen, IDH1=Isocitrate Dehydrogenase 1.

## Data Availability

The data supporting the findings of the article are available within the article.

## LIST OF ABBREVIATIONS

PA: Pilocytic astrocytoma
T1WI: T1-weighted imaging
T2WI: T2-weighted imaging
FLAIR: Fluid-attenuated inversion recovery
DWI: Diffusion-weighted imaging
ADC: Apparent diffusion coefficient
CSF: Cerebrospinal fluid
TR: Repetition time
TE: Echo time
ROIs: Regions of interest
H&E: Hematoxylin and eosin
IHC: Immunohistochemistry
ACP: Ameloblastic craniopharyngioma
AFP: Alpha-fetoprotein
HCG: Human chorionic gonadotropin
PMA: Pilomyxoid astrocytoma
GFAP: Glial fibrillary acidic protein
Olig2: Oligodendrocyte transcription factor
SYN: Synaptophysin
EMA: Epithelial membrane antigen
NeuN: Neuronal nuclear antigen
IDH1: Isocitrate dehydrogenase 1

## STANDARDS OF REPORTING

STROBE guidelines were followed.
